# Prostacyclin Synthesis and Prostacyclin Receptor Expression in the Porcine Myometrium: Prostacyclin Potential to Regulate Fatty Acid Transporters, Cytokines and Contractility-Related Factors

**DOI:** 10.3390/ani12172237

**Published:** 2022-08-30

**Authors:** Agnieszka Blitek, Mateusz Luba, Magdalena Szymanska

**Affiliations:** Institute of Animal Reproduction and Food Research, Polish Academy of Sciences, Tuwima 10, 10-748 Olsztyn, Poland

**Keywords:** pig, myometrium, the estrous cycle, pregnancy, prostacyclin, prostacyclin receptor, contractility-related factors, nutrient transporters, prostaglandin receptors, cytokines

## Abstract

**Simple Summary:**

Prostacyclin (prostaglandin I2; PGI2) is an important modulator of vascular functions and is involved in various reproductive processes. PGI2 was also described as a modulator of uterine contractility in several species, including the pig. However, its synthesis and role in the myometrium of the porcine uterus are still not fully described. The objective of this study was to evaluate profiles of PGI2 synthesis and PGI2 receptor expression in the myometrium of gilts throughout the estrous cycle and during early pregnancy and to investigate the in vitro effect of PGI2 on the mRNA expression of factors engaged in smooth muscle contraction, nutrient transport, prostaglandin synthesis and action, and inflammatory response. The obtained results showed that the synthesis of PGI2 changes in the myometrium of pigs during both the estrous cycle and early pregnancy, resulting in much greater concentrations of PGI2 in cyclic than in pregnant gilts. Moreover, PGI2 stimulated the expression of fatty acid transporters and contractility-related calponin 1 and caldesmon 1, whereas it decreased cytokine expression. This study indicates that PGI2 may participate in the regulation of myometrial functions modulating the availability of factors involved in smooth muscle activity and inflammatory reaction in the uterus of pigs.

**Abstract:**

Although prostacyclin (PGI2) has been well described as a regulator of smooth muscle activity, limited data are available concerning its role in the myometrium of pigs. The present research aimed to examine profiles of PGI2 synthase (PTGIS) and PGI2 receptor (PTGIR) expression and 6-keto PGF1α (a PGI2 metabolite) concentrations in the myometrium of gilts throughout the estrous cycle and during early pregnancy using qPCR, Western blot, and/or ELISA methods. Furthermore, myometrial explants were exposed to iloprost (a stable PGI2 analog) to investigate the effect of PGI2 on the mRNA expression of factors engaged in smooth muscle contraction, nutrient transport, prostaglandin synthesis and action, and inflammatory response. *PTGIS* mRNA expression was greater in cyclic than in pregnant gilts on days 11–12 after estrus and was accompanied by greater concentrations of 6-keto PGF1α detected in cyclic than in pregnant animals on days 11–20. Iloprost stimulated fatty acid transporters and contractility-related calponin 1 and caldesmon 1 mRNA expression and decreased interleukin 1β and tumor necrosis factor transcript abundance. The obtained results indicate a physiologically relevant role of PGI2 during the estrous cycle in the porcine myometrium with its importance for regulating the expression of contractility-, nutrient transport- and inflammatory response-related factors.

## 1. Introduction

The uterus is a complex organ composed of various cell types that form the endometrium and the myometrium. It has been well described that the successful outcome of the reproductive processes requires proper remodeling and secretory activity of the uterine endometrium [1,2,3]. However, the role of the myometrium as a local regulator of uterine functions should not be neglected. Both the contractile and secretory activities of the myometrium are important for the estrous cycle regulation and pregnancy maintenance in pigs [4,5,6]. Ovarian steroids appear as the primary modulators of myometrial function [7,8]. In addition, several endocrine and locally produced factors, including prostanoids, may act on the myometrial tissue [7,8,9,10,11].

Prostaglandin I2 (PGI2), commonly known as prostacyclin, belongs to the prostanoid family of lipid mediators. Prostanoids are derivatives of the 20-carbon omega-6 fatty acid, arachidonic acid (AA). Prostaglandin (PG) endoperoxide synthase (PGHS; generally known as cyclooxygenase) catalyzes the conversion of AA to unstable PGH2 that is subsequently transformed into PGE2, PGF2α, PGD2, PGI2, or thromboxane A2 by specific synthases [12,13]. The formation of PGI2 is catalyzed by prostaglandin I2 synthase (PTGIS), which is an enzyme located at the membrane of the endoplasmic reticulum [14]. This enzyme was primarily localized in endothelial cells, but its expression was also demonstrated in other cell types in the body [13,15,16]. PGI2 has a very short half-life in biological fluids and rapidly undergoes degradation to form an inactive 6-keto PGF1α. As a result of this, PGI2 exerts mainly auto- and paracrine actions on target cells [17,18].

The effects of PGI2 are mediated by a G protein-coupled membrane receptor, PTGIR, which possesses a typical seven transmembrane structure [17]. The linking of PGI2 to the Gs subunit of PTGIR results in the activation of adenylate cyclase and the formation of cAMP. This classical PGI2 signaling is engaged in the regulation of vascular functions and smooth muscle relaxation [13,17,19]. Mice lacking *Ptgir* expression exhibited an increased predisposition to thrombosis and impaired inflammatory response [20].

Among livestock species, profiles of PTGIS and PTGIR expression were documented in the endometrium of cyclic and/or pregnant cows [21], ewes [22], and pigs [23,24]. In the porcine endometrium, activation of PTGIR with iloprost, a stable analog of PGI2, results in an increased expression of pro-angiogenic genes [24]. PTGIR is also differentially expressed during the estrous cycle and early pregnancy in the corpus luteum of the pig and PGI2 analogs, iloprost and carbaprostacyclin, affect progesterone production in luteal cells acting through PTGIR [25]. Moreover, PGI2 may support conceptus implantation in this species because it promotes the attachment and proliferation of porcine trophoblast cells [26].

Although PGI2 has been well described as a modulator of muscle contractility [17] and the porcine myometrium is a target for PGI2 [27,28], only limited data are available regarding PGI2 synthesis and its receptor system expression in the myometrium of pigs. Moreover, the possible impact of PGI2 on the mRNA transcript abundance in the myometrial tissue has not been described for this species. Thus, the present research was undertaken to examine profiles of PTGIS and PTGIR expression and PGI2 concentration in the myometrium of cyclic and early pregnant gilts. Moreover, the effect of PGI2 on the mRNA expression of selected genes encoding proteins implicated in contractility, nutrient transport, PG synthesis and action, and/or inflammatory response was determined in vitro. In particular, gap junction protein alpha 1, calponin 1, caldesmon 1, and oxytocin receptor were chosen as contractility-related genes, while tumor necrosis factor, interleukin (IL) 1 beta, C-X-C motif chemokine ligand 8 (encodes IL8) and nuclear factor kappa B subunit 1 were selected as inflammatory response-related genes. Moreover, solute carrier family 5 member 1 and solute carrier family 38 member 1 were chosen as glucose and amino acid transporters, respectively, while CD36 and solute carrier family 27 member 4 were examined as genes encoding fatty acid transporters. Genes encoding proteins crucial for PG synthesis, namely prostaglandin-endoperoxide synthase 2, and action, i.e., PGE2 receptors type 2 and 4 and PGF receptor, were also selected for mRNA examination.

## 2. Materials and Methods

### 2.1. Animals and Sample Collection

For ex vivo analyses of myometrial PGI2 synthesis and PTGIR expression, uteri were obtained from 58 crossbred gilts (Polish Landrace x Duroc) of similar age, weight, and genetic background originating from one commercial herd. After exhibiting two estrous cycles of normal length, females were divided into two groups, cyclic and pregnant, and further observed for the next estrus behavior. Animals assigned to the cyclic group were slaughtered on days 3–4 (*n* = 6), 6–7 (*n* = 6), 11–12 (*n* = 6), 15–16 (*n* = 5) and 19–20 (*n* = 5) of their third estrous cycle. The day of the estrous cycle was confirmed by macroscopic examination of the ovaries, as outlined earlier [29]. Gilts assigned to be pregnant were bred 12 and 24 h after recognition of the estrus. The day of the second breeding was deemed the first day of pregnancy. Gilts were slaughtered on days 11–12 (*n* = 7), 15–16 (*n* = 6), 19–20 (*n* = 6), 25 (*n* = 5) and 30 (*n* = 6) of pregnancy. The day of pregnancy was verified by the morphology of conceptuses flushed from uterine horns (days 11 to 16) or by the morphology and size of embryos/fetuses attached to the uterus (days 19 to 30) [30,31]. Myometrial tissue was separated from the endometrial tissue, snap-frozen in liquid nitrogen, and maintained at −80 °C for further analyses.

For in vitro experiments, randomly selected uterine horns were collected from additional six cyclic gilts slaughtered on days 12–14 after estrus and transported to the laboratory in sterile, ice-cold PBS supplemented with antibiotics (100 IU/mL penicillin and 100 µg/mL streptomycin; P4333, Sigma-Aldrich, St. Louis, MO, USA).

### 2.2. Incubation of Myometrial Explants

In order to study the effect of PGI2 on the abundance of mRNA transcripts of selected genes, myometrial explants were used. Briefly, a 10 to 20 cm long fragment was cut off from each of six uterine horns, which were transported to the laboratory, and opened longitudinally. Then, the myometrial tissue was separated from the endometrium using scissors, washed with sterile PBS, and cut with a scalpel blade into small pieces (20 to 25 mg). A total of 110–120 mg of the myometrial tissue was placed into glass vials (5 explants per vial) containing 2 mL of incubation medium (Dulbecco’s Modified Eagle’s Medium [D2902, Sigma-Aldrich] supplemented with antibiotics and 25 mM HEPES [H4034, Sigma-Aldrich]) and pre-incubated for 2 h. Then, the pre-incubation media were removed, and myometrial explants were incubated for 8 h in an incubation medium enriched with 0.1% BSA (81-003-3, Millipore, Kankakee, IL, USA) with vehicle (0.01% ethanol; control) or with 1 µM of iloprost (a stable analog of PGI2; 18215, Cayman Chemicals, Ann Arbor, MI, USA). After incubation, myometrial explants were washed with ice-cold PBS, snap-frozen in liquid nitrogen, and maintained at −80 °C until RNA isolation. The treatment was carried out in duplicate using myometrial tissue from six gilts.

### 2.3. Total RNA Isolation and Real-Time PCR

Total RNA was extracted from the myometrial tissue collected at slaughter and from myometrial explants after incubation using a Total RNA Mini kit (A&A Biotechnology, Gdansk, Poland) and treated with DNase I (Sigma-Aldrich) in accordance with the manufacturer’s instructions. RNA samples (1 µg) were reverse transcribed using a High Capacity cDNA Reverse Transcription kit (Applied Biosystems, Waltham, MA, USA) according to the manufacturer’s protocol.

Diluted cDNA from RT-PCR was used to analyze the mRNA transcript abundance of selected genes with an ABI Viia7 Sequence Detection System (Life Technologies Inc., Carlsab, CA, USA). In order to determine the mRNA expression of genes: *PTGIS*, *PTGIR*, *GJA1*, *OXTR*, *CNN1*, *CALD1*, *SLC5A1*, *SLC38A1*, *CD36*, *SLC27A4*, *PTGS2*, *PTGER2*, *PTGER4*, *PTGFR*, *TNF*, *IL1B*, *CXCL8*, *NFKB1*, *GAPDH*, *HPRT1*, and *ACTB*, 15 ng of cDNA was amplified using TaqMan Gene Expression Assays (Applied Biosystems). All abbreviations of the studied genes, their full names, and the ID numbers of the TaqMan probes are outlined in Table 1. Each PCR reaction (10 μL) was carried out in duplicates in a 384-well plate using the following conditions: initial denaturation for 10 min at 95 °C, followed by 45 cycles of 15 s of denaturation at 95 °C and then 60 s of annealing at 60 °C. The control reactions in the absence of reverse transcriptase were conducted to test for genomic DNA contamination. For both RT-PCR and qPCR, no template controls with nuclease-free water were performed to check for possible reagent contamination. Data from real-time PCR were analyzed using the PCR Miner algorithm [32], as previously described [33]. In order to select the most stable reference genes among *GAPDH*, *HPRT1*, and *ACTB*, NormFinder software was applied [34]. All expression data for each target gene were normalized against geometric averaging of *GAPDH* and *HPRT1*.

### 2.4. Preparation of Myometrial Tissue Homogenates

Myometrial tissue of cyclic and pregnant gilts was homogenized utilizing an ice-cold homogenization buffer (50 mM Tris-HCl, pH 8.0; 150 mM NaCl, 1% Triton X-100, 1 mM EDTA) supplemented with 10 µL/mL Protease Inhibitor Cocktail (P8340; Sigma-Aldrich) and centrifuged for 10 min at 800× *g* at 4 °C. Subsequently, a portion (0.2 µL) of each supernatant was collected, stored at −80 °C, and used for Western blot analysis of PTGIS and PTGIR proteins. The rest of the supernatants were centrifuged for an additional 1 h at 100,000× *g* at 4 °C to obtain cytosolic fraction, which was used for enzyme-linked immunosorbent assay (ELISA) of 6-keto PGF1α. Total protein concentrations in tissue homogenates were further determined [35].

### 2.5. Western Blot Analysis

Myometrial tissue samples (10 µg for both PTGIS and PTGIR) were dissolved in SDS gel-loading buffer (50 mM Tris-HCl, pH 6.8; 4% SDS, 20% glycerol, and 2% β-mercaptoethanol), heated to 95 °C for 5 min, and separated on 8% (for PTGIS) or 10% (for PTGIR) SDS-PAGE. Separated proteins were electroblotted onto 0.45 µm pore size polyvinylidene difluoride membrane in transfer buffer (20 mM Tris-HCl, pH 8.2; 150 mM glycine, 20% methanol). Then, nonspecific binding sites were blocked using 5% nonfat dry milk in TBS-T (Tris-buffered saline, containing 0.1% Tween-20) for 1.5 h at room temperature. After blocking, membranes were incubated overnight with primary antibodies: rabbit polyclonal to PTGIS (dilution 1:100; 160640, Cayman Chemicals) or rabbit polyclonal to PTGIR (1:200 dilution; 10005518, Cayman Chemicals) at 4 °C. After that, membranes were washed with TBS-T buffer and incubated with anti-rabbit IgG-alkaline phosphatase antibody (1:20,000 dilution; A8025; Sigma-Aldrich) at room temperature for 1 h. Immune complexes were visualized using a standard alkaline phosphatase visualization procedure. All images were captured with ChemiDoc MP Imaging System (Bio-Rad, Hercules, CA, USA), and protein bands were quantitated using Image Lab 6.0 Software (Bio-Rad). As internal controls for protein loading, rabbit polyclonal to ACTB antibody (1:3000 dilution; ab8227, abcam, Cambridge, UK; for PTGIS) or rabbit polyclonal to GAPDH antibody (1:2000 dilution; ab9485, abcam; for PTGIR) were used.

### 2.6. ELISA of 6-Keto PGF1α

In order to analyze concentrations of PGI2 metabolite in myometrial tissue homogenates, a 6-keto PGF1α ELISA kit (515211; Cayman Chemicals) was used according to the manufacturer’s instructions. Myometrial tissue samples were diluted 1:400 before the assay. The sensitivity of this assay was 1.6 pg/mL, and intra- and inter-assay coefficients of variation were 7.8% and 9.1%, respectively. Levels of 6-keto PGF1α in the myometrium were standardized per total protein content.

### 2.7. Statistical Analysis

All statistical analyses were conducted using GraphPad PRISM v. 9.3.1 (GraphPad Software, Inc., San Diego, CA, USA). In order to test the profiles of PTGIS and PTGIR mRNA and protein expression and the concentrations of 6-keto PGF1α in the myometrium of cyclic and pregnant gilts, two-way ANOVA followed by Tukey’s post hoc test was used. This analysis included the effect of the day after estrus and the reproductive status of animals (cyclic vs. pregnant). In order to evaluate the effect of iloprost on the mRNA transcript abundance of selected genes, a paired *t*-test was used. All numerical data are presented as the mean ± SEM. Means were considered to be statistically different at *p* ≤ 0.05, with a tendency for significance at *p* ranging from 0.06 to 0.07.

## 3. Results

### 3.1. PTGIS mRNA and Protein Expression and 6-Keto PGF1α Concentrations in the Myometrial Tissue of Cyclic and Early Pregnant Gilts

**Figure 1:**
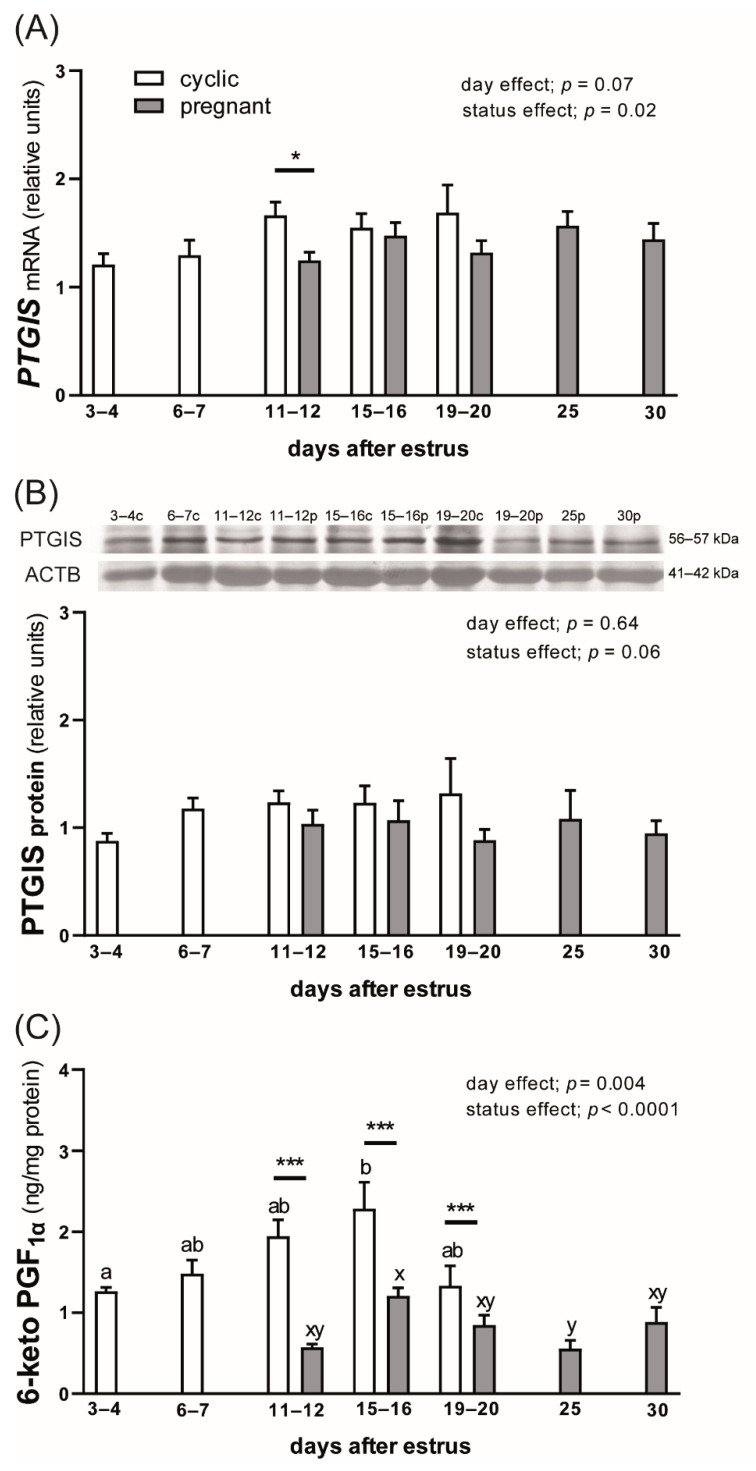
Expression of prostaglandin I2 (PGI2; prostacyclin) synthase (PTGIS) mRNA (**A**) and protein (**B**) and concentrations of 6-keto PGF1α (a PGI2 metabolite; **C**) in the myometrium of cyclic and early pregnant gilts. Values from real-time PCR for *PTGIS* were normalized to geometric averaging of *GAPDH* and *HPRT1* gene expression. Values from densitometric analyses of PTGIS protein were normalized to ACTB. Representative blots are presented (c—cyclic, p—pregnant; original western blots are included in Figure S1). Data are expressed as the mean ± SEM (*n* = 5–7). Bars marked with various letters (ab—for cyclic gilts; xy—for pregnant gilts) are different among groups. Asterisks specify differences between cyclic and pregnant animals on particular days after estrus (* *p* < 0.05; *** *p* < 0.001).

PTGIS protein showed stable profiles of expression in the myometrial tissue during examined periods of both the estrous cycle and early pregnancy (*p* = 0.64; Figure 1B), while the day after estrus tended to affect *PTGIS* mRNA expression (*p* = 0.07; Figure 1A). Moreover, the reproductive status of animals affected the abundance of *PTGIS* transcripts (*p* = 0.02; Figure 1A). On days 11–12 after estrus, gilts from the pregnant group showed lower expression of *PTGIS* mRNA compared with cyclic animals (*p* < 0.05). Moreover, a tendency to decrease PTGIS protein levels in pregnant gilts compared with cyclic animals was detected (*p* = 0.06; Figure 1B).

Concentrations of 6-keto PGF1α in the cytosolic fraction of the myometrial tissue were affected by both the day after estrus (*p* = 0.004) and by the reproductive status of animals (*p* < 0.0001; Figure 1C). During the estrous cycle, the content of 6-keto PGF1α in the myometrium increased between days 3–4 and 15–16 (1.26 ± 0.05 vs. 2.14 ± 0.36 ng/mg protein; *p* < 0.05). During pregnancy, a decrease in the concentration of 6-keto PGF1α was detected on day 25 compared with days 15–16 (0.56 ± 0.1 vs. 1.21 ± 0.36 ng/mg protein; *p* < 0.01). The effect of the reproductive status on the content of PGI2 metabolite in the myometrial tissue was observed on days 11 to 20 after estrus when concentrations of 6-keto PGF1α were lower in pregnant than in cyclic gilts (*p* < 0.001).

### 3.2. PTGIR mRNA and Protein Expression in the Myometrial Tissue of Cyclic and Early Pregnant Gilts

**Figure 2:**
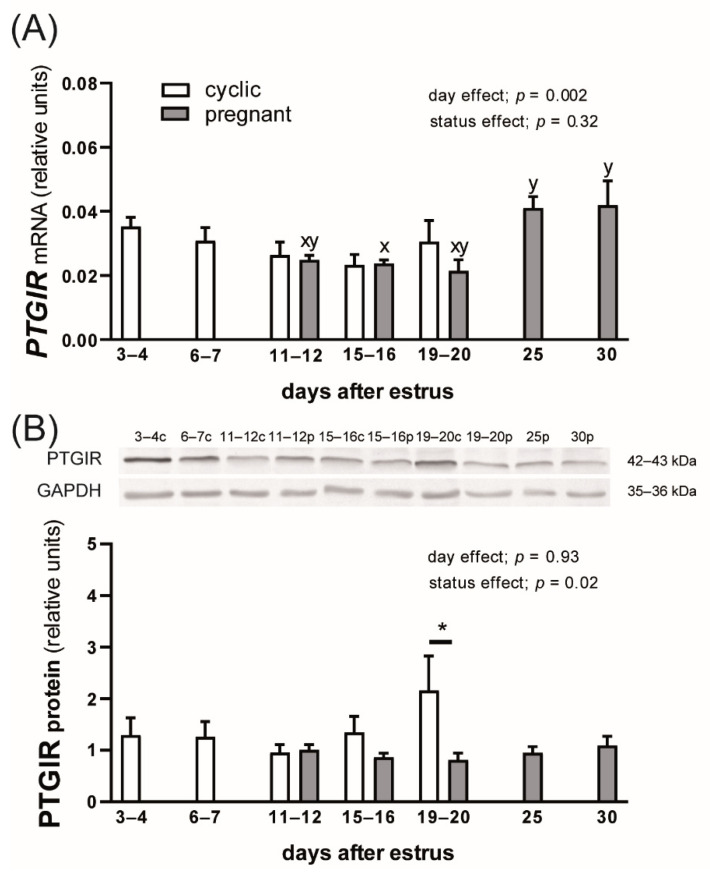
Prostaglandin I2 receptor (PTGIR) mRNA (**A**) and protein (**B**) expression in the myometrium of cyclic and early pregnant gilts. Values from real-time PCR for *PTGIR* were normalized to geometric averaging of *GAPDH* and *HPRT1* gene expression. Values from densitometric analyses of PTGIR protein were normalized to GAPDH. Representative blots are presented (c—cyclic, p—pregnant; original western blots are included in Figure S1). Data are expressed as the mean ± SEM (*n* = 5–7). Bars marked with various letters are different among the group of pregnant females. Asterisk specifies the difference between cyclic and pregnant animals on days 19–20 after estrus (* *p* < 0.05).

The day after estrus but not the reproductive status of animals affected *PTGIR* mRNA expression in the porcine myometrium (*p* = 0.002 and *p* = 0.32, respectively; Figure 2A). A greater abundance of *PTGIR* transcripts was detected in the myometrial tissue on days 25 and 30 compared with days 15–16 of gestation (*p* < 0.05). PTGIR protein level was affected by the reproductive status of animals (*p* = 0.02) but not by the day after estrus (*p* = 0.93; Figure 2B). On days 19–20 after estrus, pregnant gilts showed lower concentrations of PTGIR protein compared with cyclic animals (*p* < 0.05).

### 3.3. Effect of Iloprost on the mRNA Transcript Abundance of Selected Genes in Myometrial Explants

**Figure 3:**
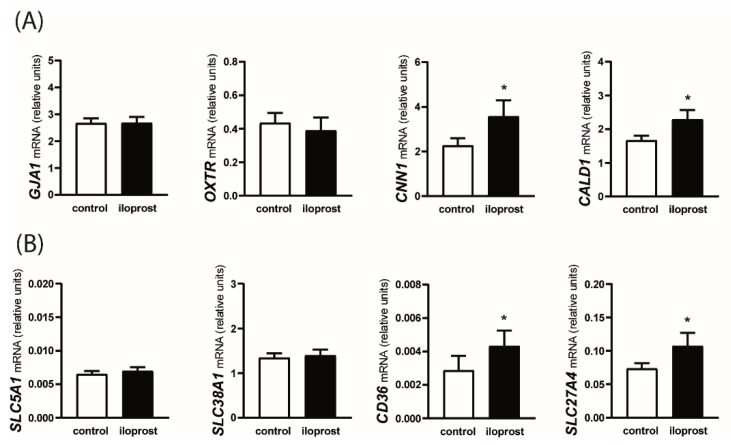
Effect of prostaglandin I2 (PGI2; prostacyclin) analog, iloprost, on the mRNA expression of contractility-related genes (panel **A**; *GJA1, OXTR, CNN1,* and *CALD1*) and genes encoding transporters (panel **B**) of glucose (*SCL5A1*), amino acids (*SLC38A1*), and fatty acids (*CD36* and *SLC27A4*) in the myometrium. Myometrial explants were incubated for 8 h without (control) or with 1 µM of iloprost. Values from real-time PCR for each studied mRNA transcript were normalized to geometric averaging of *GAPDH* and *HPRT1* mRNA expression. Data are expressed as the mean ± SEM (*n* = 6). Asterisk specifies the difference compared with the control value (* *p* < 0.05).

The treatment of myometrial explants with iloprost increased the mRNA expression of *CNN1* and *CALD1* (*p* < 0.05) but did not affect the relative abundance of *GJA1* and *OXTR* transcripts (Figure 3A). Among nutrient transporter encoding genes, iloprost stimulated the mRNA expression of *CD36* and *SLC27A4* (*p* < 0.05), while the expression of *SLC5A1* and *SLC38A1* mRNA did not differ between control and iloprost-treated myometrial explants (Figure 3B).

**Figure 4:**
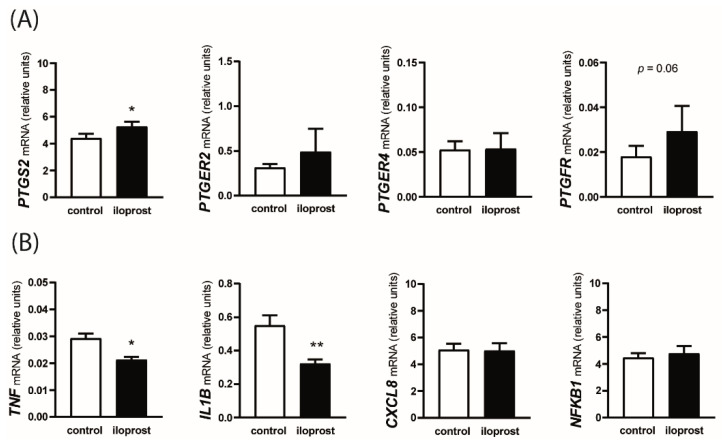
Effect of prostaglandin I2 (PGI2; prostacyclin) analog, iloprost, on the mRNA expression of genes involved in PG synthesis (panel **A**; *PTGS2*) and action (panel **A**; *PTGER2*, *PTGER4*, and *PTGFR*) and genes involved in the inflammatory response (panel **B**; *TNF, IL1B, CXCL8,* and *NFKB1*) in the myometrium. Myometrial explants were incubated for 8 h without (control) or with 1 µM of iloprost. Values from real-time PCR for each studied mRNA transcript were normalized to geometric averaging of *GAPDH* and *HPRT1* mRNA expression. Data are expressed as the mean ± SEM (*n* = 6). Asterisks specify the difference compared with the control value (* *p* < 0.05; ** *p* < 0.01).

As shown in Figure 4A, the addition of iloprost to the incubation medium resulted in a greater expression of *PTGS2* mRNA (*p* < 0.05) and tended to increase *PTGFR* mRNA expression (*p* = 0.06). The relative abundance of *PTGER2* and *PTGER4* transcripts were not affected by the presence of iloprost. Among inflammatory response-related genes, iloprost decreased *TNF* and *IL1B* mRNA expression (*p* < 0.05 and *p* < 0.01, respectively) and had no effect on *CXCL8* and *NFKB1* mRNA expression (Figure 4B).

## 4. Discussion

To the best of our knowledge, the present study is the first to demonstrate profiles of PGI2 concentrations and PGI2 receptor expression throughout the estrous cycle and during the peri-implantation period in the myometrium of livestock species. The results of the present study revealed increased synthesis of PGI2 and the expression of its membrane receptor in the myometrium of cyclic compared with pregnant gilts. Moreover, PGI2 was shown as a modulator of the myometrial expression of contractility-related genes, fatty acid transporters, PG synthesis enzymes, and cytokines.

The expression of the terminal enzyme of the PGI2 synthesis pathway, PTGIS, was shown in the myometrium of pregnant and/or non-pregnant sheep [22,36] and humans [37,38]. Moreover, PTGIS protein was localized in the smooth muscle cells of the porcine myometrium [28]. The results of the present study revealed that PTGIS was abundantly expressed throughout the estrous cycle and during early pregnancy in the myometrial tissue. However, PTGIS mRNA and protein expression did not vary significantly on particular days of the estrous cycle or gestation. Interestingly, *PTGIS* mRNA transcript abundance was greater, and PTGIS protein content tended to be greater in cyclic than in pregnant gilts. Steady-state levels of PTGIS expression during examined periods were accompanied by dynamic changes in 6-keto PGF1α concentration detected in the myometrial tissue throughout the estrous cycle and during early pregnancy. Thus, elevated content of PGI2 in the myometrial tissue of cyclic gilts results from a greater PTGIS activity rather than a greater abundance of this enzyme.

Increased myometrial synthesis of PGI2 during the estrous cycle as compared with respective days of pregnancy observed in the present study seems somewhat surprising because PGI2 is a well-known relaxant of smooth muscles [17,39], and the quiescent uterus is important for developing embryos/fetuses. In the myometrium of pregnant rats, the most intense synthesis of PGI2 was detected in the area remarkably stretched by the growing embryos, and spontaneous contractility was decreased in regions with a lower myometrial synthesis of PGI2 [40]. Thus, we expected that in pigs, elevated synthesis of PGI2 during the peri-implantation period would occur as part of some local changes ensuring the most favorable conditions for implanting conceptuses. The present results, however, showed a substantial increase in 6-keto PGF1α concentrations on days 15–16 of the estrous cycle compared with days 3–4 after estrus. Moreover, much greater concentrations of PGI2 metabolite were detected on days 11 to 20 of the estrous cycle compared with respective days of pregnancy. These results indicate a role of PGI2 in the porcine myometrium during luteolysis and pre-ovulatory period rather than during early pregnancy. Because much more attention has been attributed to the role of PGI2 in the myometrium during late pregnancy and labor [38,39,41] than during the estrous or menstrual cycle, it is difficult to assess whether increased synthesis and/or accumulation of PGI2 in this tissue during the late luteal phase is peculiar only to pigs. On the other hand, a similar phenomenon was described for women because PGI2 concentrations increase during late pregnancy and PGI2 is the most prominent PG at the time of parturition, while PGI2 shows relaxant effects on the myometrium during labor (for review: [7]).

PGI2 acts on target cells primarily via activation of its specific membrane receptor, PTGIR [17]. As we demonstrated in the present study, *PTGIR* mRNA level did not change during the estrous cycle but increased on days 25 and 30 of gestation compared with days 15–16. PTGIR protein level did not vary during both studied periods after estrus, but similar to PGI2 metabolite, it was greater in cyclic than in pregnant gilts on days 19–20 after estrus. Our present results confirm previous data of Jana and co-workers [28], who localized PTGIR protein in the muscular and vascular cells of the porcine myometrium but are the first to show expression patterns of myometrial PTGIR during the estrous cycle and early pregnancy in pigs. Similar to data on PTGIS expression, only limited papers are available regarding PGI2 receptor expression in the uterine myometrium. PTGIR protein was localized in myometrial smooth muscle cells in non-pregnant [42] and pregnant [43] women. Moreover, PGI2 binding sites, along with a possible relaxing effect of PGI2 on the uterine vasculature, were reported for non-pregnant bovine myometrial tissue [44]. Greater content of PTGIR protein in the myometrium of cyclic than pregnant gilts observed in the current study on days 19–20 points to a possible role of PGI2 as a modulator of uterine motility during the peri-ovulatory period.

In order to determine the possible role of PGI2 in the porcine myometrium, tissue explants were exposed to iloprost, a stable analog of PGI2, and examined for mRNA transcript abundance of selected genes; in particular, genes involved in contractility, nutrient transport, inflammatory response, and PG synthesis and action were studied. These processes were previously reported as important for the proper function of the myometrium [7,8,10,11,45].

The activation of the myometrial tissue depends on the coordinated expression of several contraction-related proteins, including connexin 43 (encoded by *GJA1*) and oxytocin [7]. Both proteins are important for myometrial contractility during labor and delivery in women [8]. Moreover, connexin 43 and oxytocin receptors were detected in the myometrium of cyclic and/or pregnant gilts [46,47], and oxytocin was shown as an effective stimulator of the myometrial contractility during luteolysis [48]. Of interest, PGI2 stimulated connexin 43 expression and enhanced oxytocin-induced contractility in human myometrial tissue [41]. Our present results, however, showed that the PGI2 analog did not affect *GJA1* and *OXTR* mRNA expression in the myometrium of pigs. It indicates that PGI2 does not participate in the preparation of myometrial tissue for oxytocin-inducing contractions before luteolysis in this species.

Smooth muscle contractions result from actin and myosin interactions. Moreover, thin filament-related proteins, calponin and caldesmon, modulate the actomyosin complex [49]. In the current experiment, the mRNA expression of both *CNN1* and *CALD1* was stimulated by iloprost. These results are consistent with previously demonstrated greater expression of calponin and caldesmon mRNA and protein in human myometrial tissue exposed to iloprost [41]. Although Fetalvero and co-workers [41] suggested a role for both proteins as important for the contractile response of the myometrium during initiation and progression of the parturition, calponin and caldesmon inhibit the activity of actin-induced myosin adenosine triphosphatase and were proposed as inhibitory regulators of smooth muscle contractions [50,51]. Thus, iloprost-induced myometrial *CNN1* and *CALD1* mRNA expression observed in the present study point to relaxant rather than contractile action of PGI2 in this tissue. A possible dual role for PGI2 was suggested in the myometrium of women, including myometrial quiescence and activation [52]. Accordingly, both inhibitory and stimulatory effects of PGI2 on the myometrial contractility in cyclic gilts were reported [27,28].

Cellular growth and activity require the supply of various nutrients. Myometrial smooth muscle cells use both glucose and fatty acids as major energy substrates [45]. Moreover, amino acids are important for the proper functioning of the myometrium [53]. Increased amino acid catabolism and fatty acid oxidation were observed in the myometrium during labor in women [54]. A crucial rate-limiting process in glucose, fatty acids, and amino acid utilization involves their transport across the cell membrane. A number of glucose and amino acid transporters, including SLC5A1 and SLC38A1, respectively, were reported in the endometrium of the porcine uterus [55]. The results of the present research showed, for the first time, the mRNA expression of both *SLC5A1* and *SLC38A1* in the porcine myometrium and demonstrated that their abundance in this tissue did not change in response to iloprost. Similarly, another analog of PGI2, carbaprostacyclin, did not affect the expression of glucose and amino acid transporters in porcine endometrial cells [56]. Of interest is the addition of iloprost stimulated *CD36* (also known as fatty acid translocase; FAT/CD36) and *SLC27A4* mRNA expression in the myometrial explants (present data). These results indicate that PGI2 may support fatty acid delivery into the myometrial cells to enhance the energy status of this tissue.

Endometrium-derived PGE2 and PGF2α acting via their membrane receptors, PTGER and PTGFR, respectively, regulate various reproductive events during the estrous cycle and early gestation [3,57]. The uterine myometrium also synthesizes both PGs and expresses their receptors [7,9,10]. In the myometrium of pigs, PTGER2 was shown as a relaxant, while PTGFR was described as a contractile receptor [58]. In the current study, iloprost did not affect *PTGER2* and *PTGER4* mRNA expression but tended to increase *PTGFR* transcript abundance. It indicates that PGI2 may affect the sensitivity of the myometrium to contractile PGF2α; however, this suggestion requires further research. PGHS2 protein (encoded by *PTGS2* gene), the rate-limiting enzyme in PG synthesis, was detected in the myometrium of gilts [58], and its expression increases in response to oxytocin [59]. As we showed in the present study, *PTGS2* mRNA transcript abundance in myometrial explants was stimulated by iloprost treatment. It points to the possible function of PGI2 in the sustained generation of PGs in this tissue.

The intense remodeling of the endometrium occurring during each estrous cycle involves the modulation of synthesis and/or secretion of various cytokines and inflammatory mediators [11]. As we demonstrated here, iloprost inhibited *IL1B1* and *TNF* mRNA expression in the porcine myometrium. Such a result is consistent with the previously suggested anti-inflammatory action of PGI2 since iloprost suppressed pro-inflammatory cytokine expression in monocytes and dendritic cells [60,61,62]. Enhanced inflammatory response and increased pro-inflammatory cytokine content play a role in the transformation of the myometrial tissue from a quiescent to a contractile state before parturition [8,63]. Therefore, the inhibitory effect of iloprost on *IL1B1* and *TNF* transcript abundance in the porcine myometrium may indicate that PGI2 alleviates pro-inflammatory reactions in the myometrium and protects the uterus from excessive contractility. However, this hypothesis requires further verification.

## 5. Conclusions

In summary, this is the first demonstration of PTGIS expression and PGI2 concentration profiles in the porcine myometrium during the estrous cycle and early pregnancy. Increased synthesis of PGI2 on days 11 to 20 of the estrous cycle compared with respective days of pregnancy indicates a physiologically relevant role of PGI2 in the porcine myometrium during the luteolysis and pre-ovulatory period. Moreover, our data showed day- and reproductive status-dependent changes in the abundance of PGI2 receptor transcripts in the myometrium of pigs. Furthermore, a stable agonist of PGI2 stimulated mRNA expression of thin-filament-related proteins, fatty acid transporters, and PG synthesis enzyme and decreased the mRNA expression of cytokines in the myometrial explants in vitro. Therefore, in addition to the direct regulatory action of PGI2 on myometrial contractility [27,28], this prostanoid may modulate the availability of factors involved in smooth muscle activity and inflammatory reaction (present data) in the uterus of pigs.

Our findings broaden the knowledge about the significance of PGI2 in the reproductive tract of pigs and provide valuable insights into the mechanisms regulating myometrial function. Uterine motility is important for the secretory activity of the endometrium during luteolysis, as well as for gamete and embryo transfer in the uterine lumen. Identification of factors affecting the secretory and contractile functions of the myometrium would be of great benefit for the control of pig reproduction. Therefore, further, more extensive studies are required to evaluate the importance of intense PGI2 synthesis during the estrous cycle in pigs.

## Figures and Tables

**Table 1:** Full names of genes exploited to examine the mRNA transcript abundance with real-time PCR.

Abbreviation	Gene Name	ID of TaqMan Probe
*PTGIS*	Prostaglandin I synthase	Ss03374149_m1
*PTGIR*	Prostaglandin I receptor	- ^1^
*GJA1*	Gap junction protein alpha 1	Ss03375693_u1
*OXTR*	Oxytocin receptor	Ss03382363_u1
*CNN1*	Calponin 1	Ss03392449_g1
*CALD1*	Caldesmon 1	Ss06890042_m1
*SLC5A1*	Solute carrier family 5 member 1	Ss03374375_ml
*SLC38A1*	Solute carrier family 38 member 1	- ^1^
*CD36*	CD 36 molecule	Ss03388549_m1
*SLC27A4*	Solute carrier family 27 member 4	Ss04329560_m1
*PTGS2*	Prostaglandin endoperoxide synthase 2	Ss03394692_m1
*PTGER2*	Prostaglandin E receptor 2	Ss03374177_g1
*PTGER4*	Prostaglandin E receptor 4	Ss03377412_u1
*PTGFR*	Prostaglandin F receptor	Ss03393819_s1
*TNF*	Tumor necrosis factor	Ss03391318_g1
*IL1B*	Interleukin 1 beta	Ss04321151_m1
*CXCL8*	C-X-C motif chemokine ligand 8	Ss03392437_m1
*NFKB1*	Nuclear factor kappa B subunit 1	Ss03388579_m1
*GAPDH*	Glyceraldehyde-3-phosphate dehydrogenase	Ss03375435_u1
*HPRT1*	Hypoxanthine phosphoribosyltransferase 1	Ss03388274_m1
*ACTB*	Actin beta	Ss03376081_u1

^1^ Designed by Applied Biosystems, Thermo Fisher Scientific; for *PTGIR*: GeneBank accession no. NC_010448.3; for *SLC38A1*: GeneBank accession no. NC_010447.5.

## Data Availability

Not applicable.

## Supplementary Materials

The following are available online at https://www.mdpi.com/article/10.3390/ani12172237/s1, Figure S1: Blots used for quantitative analyses of respective bands.
