# Supplementary material for: Prostacyclin Synthesis and Prostacyclin Receptor Expression in the Porcine Myometrium: Prostacyclin Potential to Regulate Fatty Acid Transporters, Cytokines and Contractility-Related Factors
Source: Animals (Basel). 2022 Aug 30;12(17):2237. doi: 10.3390/ani12172237 (PMC9454576; doi:10.3390/ani12172237)

Blitek et al., *Prostacyclin synthesis and prostacyclin receptor expression in the porcine myometrium; prostacyclin potential to regulate fatty acid transporters, cytokines, and contractility-related factors*

Supporting information Figure S1: blots used for quantitative analyses of respective bands

1. Explanation of abbreviations used below in blots:
dc, day of the estrous cycle
dp, day of pregnancy

2. We would like to explain that „control sample" refers to the sample that has been separated in each gel and used for calculation of relative units for all samples.
Moreover, „random sample" means that some randomly chosen samples were applied on the gel in one or two marginal paths to avoid excessive bending of bands at the edge of gels; these samples were not included in band intensity calculations

3. Red color frames in one blot for PTGIS and one blot for PTGIR were chosen as representative blots and presented in Figures 1 and 2, respectively.

PTGIS protein

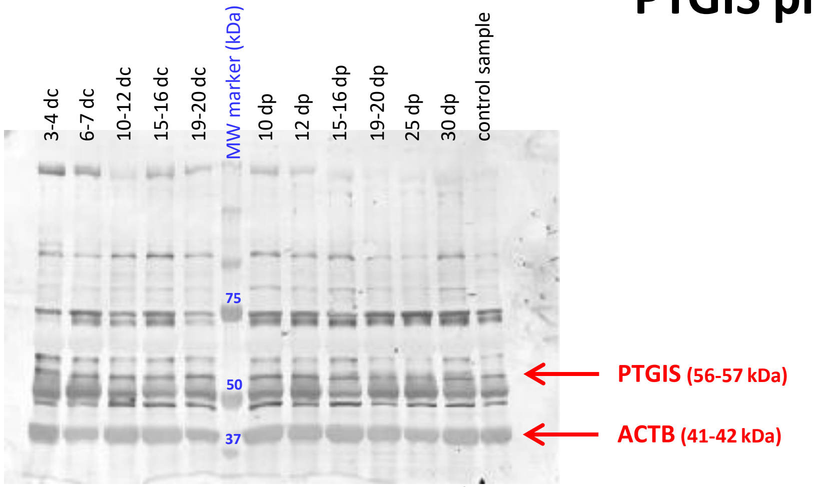

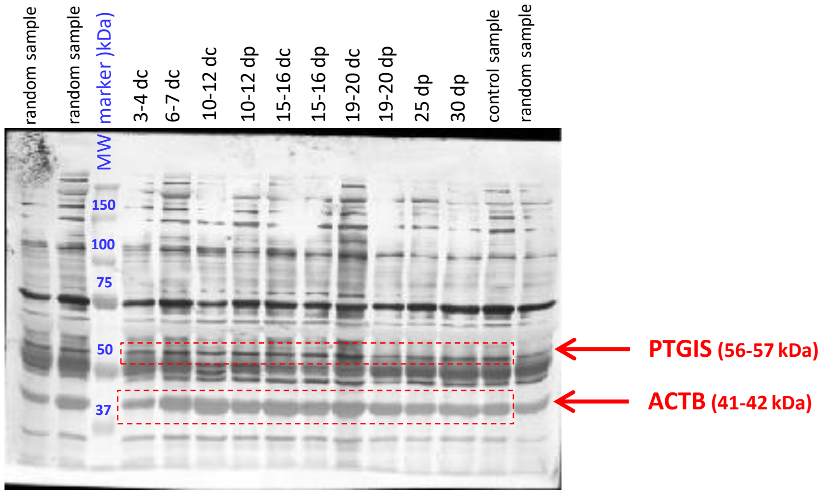

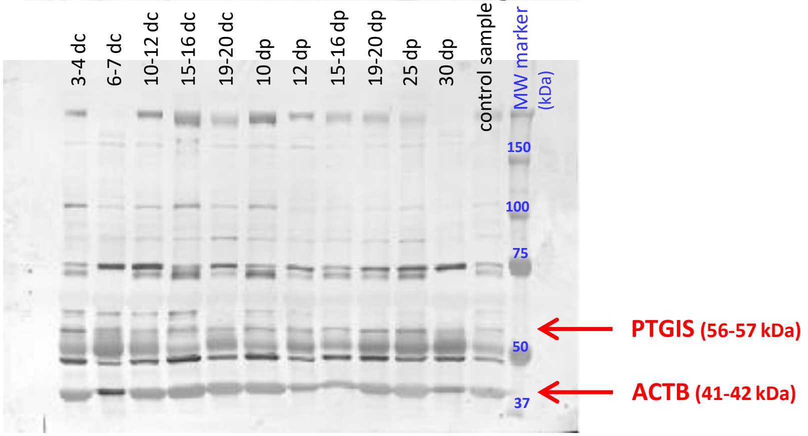

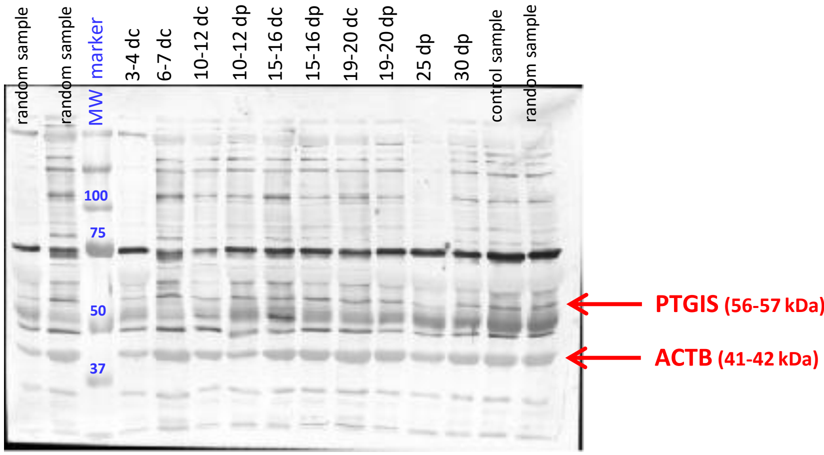

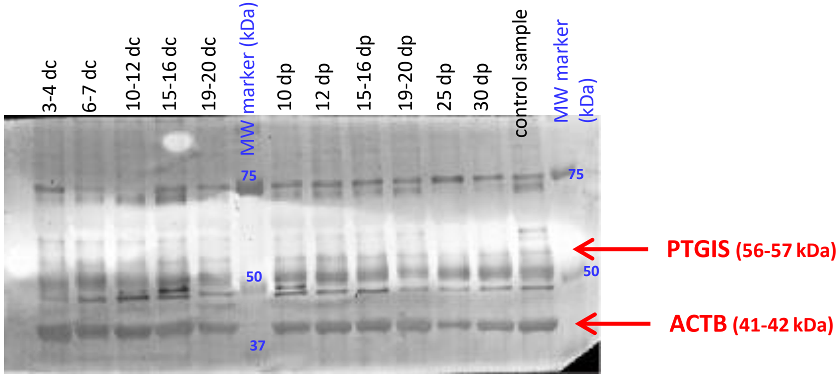

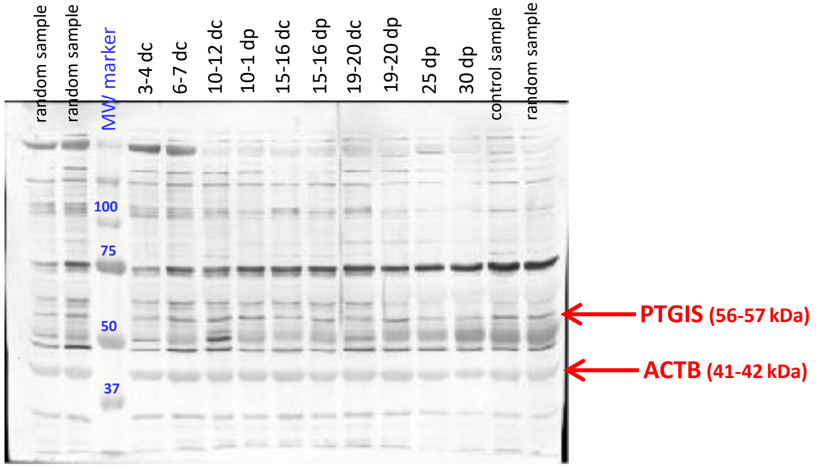

PTGIR protein

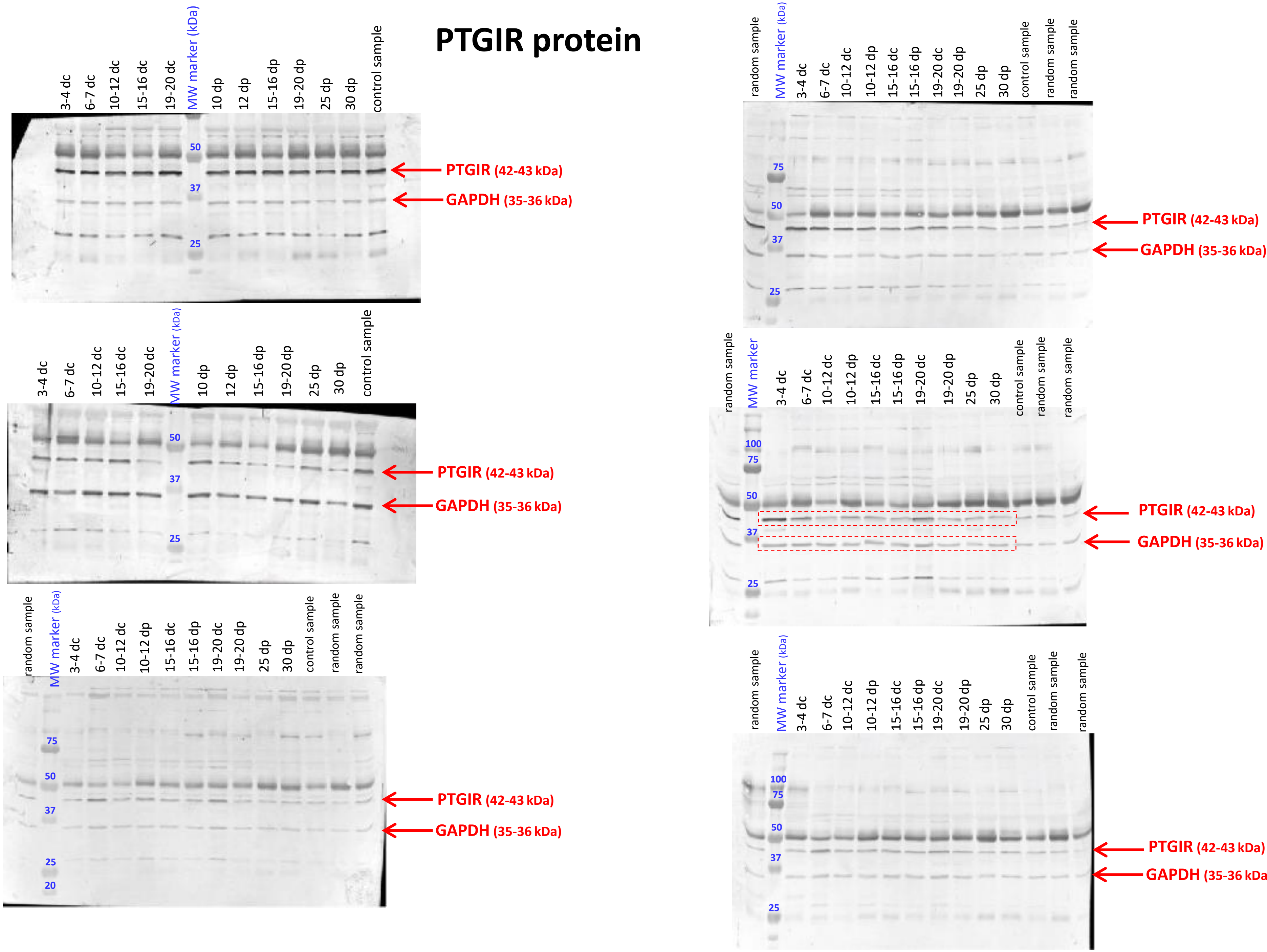

Supplement: Supplementary file 1 [file animals-12-02237-s001.zip › animals-1863131-supplementary.pdf]
